# Multi-center prospective study on central line-associated bloodstream infections in 79 ICUs of China

**DOI:** 10.1186/s12879-021-06871-5

**Published:** 2021-12-04

**Authors:** Cui Zeng, Anhua Wu, Liuyi Li, Huixue Jia

**Affiliations:** 1 Teaching and Research Section of Clinical Nursing, Xiangya Hospital, Central South University, Changsha, China; 2 Department of Infection Control, Xiangya Hospital, Central South University, Changsha, China; 3 Department of Infection Control, Peking University First Hospital, Beijing, China

**Keywords:** Central venous catheter, Central line associated blood stream infection, Intensive care unit, Surveillance, Epidemiology

## Abstract

**Background:**

China has not yet established a national surveillance network such as NHSN from America, so there is still no large-scale investigations on central line-associated bloodstream infection (CLABSI) incidence. Several retrospective studies in China reported that the incidence of CLABSI varied due to inconsistent diagnostic criteria. We performed a nationwide survey to investigate the utilization rate of central venous catheters (CVCs) and the incidence of CLABSI in ICUs of different areas of China.

**Methods:**

This is a prospective multi-center study. Patients admitted to ICUs with the use of CVCs between January 1, 2014 and December 31, 2018 were enrolled in this study. Hospitals were given the definition of catheter-related bloodstream infection as: a laboratory-confirmed bloodstream infection where CVC was in place on the date of event or the day before. The characteristics of patients, information of catheterization, implementation rates of precautions, and CLABSIs were collected. The statistical analysis was performed by SPSS 25.0 software and website of Open Source Epidemiologic Statistics for Public Health.

**Results:**

A total of 38,212 patients and 466,585 catheter days were involved in surveillance. The average CLABSI incidence in a thousand catheter days was 1.50, the lowest incidence unit was in pediatric ICU (0/1000 catheter days), and the lowest incidence area was in Northeast China (0.77/1000 catheter days), while the highest incidence unit was in cardiac ICU (2.48/1000 catheter days) and the highest incidence area was in Eastern China (1.62/1000 catheter days). The average utilization rate of CVC was 42.85%, the lowest utilization rate was in pediatric ICU (5.85%) and in Central China (38.05%), while the highest utilization rate was in surgical ICU (64.92%) and in Western China (51.57%). Among the 702 CLABSI cases reported, a total of 735 strains of pathogens were cultured. *Staphylococcus* spp. was the most common organism isolated (27.07%), followed by *Enterobacteriaceae* (22.31%). The implementation rates of all precautions showed an upward trend during the study period (*P* ≤ 0.001).

**Conclusion:**

The average incidence of CLABSI in ICUs in China is 1.5/1000 catheter days, similar to the rates reported in developed countries but lower than previous reports in China. CLABSI incidence showed regional differences in China. It is necessary to implement targeted surveillance of CLABSI cases by using standardized CLABSI surveillance definitions and methodologies.

## Background

With the development of medical science and technology, various vascular catheters have been widely used in clinical practice, not only in critical wards and hemodialysis departments, but also in general departments, mainly for infusion, chemotherapy, hemodialysis, parenteral nutrition support and hemodynamic surveillance [1]. However, the wide use of catheters increases the risk of catheter-related local and bloodstream infections in patients. Mortality induced by central line-associated bloodstream infection (**CLABSI**) could be as high as 28–30% [2].

Several retrospective studies in China reported that the incidence of CLABSI varied from 1.67 to 16.96 per 1000 catheter days [3–5], and most common pathogens associated with CLABSI were Gram-positive bacteria [6, 7]. The wide range of the reported incidences mainly caused by the inconsistent diagnostic criteria and different capacity of diagnosis. To our knowledge, there is still no large-scale investigations on CLABSI incidence in mainland China, due to the difficulties in data collection and low compliance of surveillance. Therefore, we performed a nationwide survey of CLABSI incidence in intensive care unit (ICU) to evaluate the current status in China and develop strategies to reduce CLBASI rates.

## Methods

### Study design

This study conducted a prospective surveillance of CLABSI infection in ICU through multi-center cooperation.

### Site of study

Data collection was based on province/municipality as a basic unit, and the participating hospitals and ICUs had changed slightly every year during the study.

Totally 79 ICUs (50 General ICUs, 6 Emergency ICUs, 6 Surgical ICUs, 5 Neurological ICUs, 4 Respiratory ICUs, 4 Medical ICUs, 2 Coronary Care Units, 2 Pediatric ICUs) from 62 hospitals in ten provinces (Liaoning, Shandong, Henan, Shanxi, Jiangsu, Zhejiang, Guangdong, Guizhou, Sichuan, Hunan) and one municipality (Beijing) participated in this study. Only one of the 62 hospitals in this survey is a special hospital (Maternal and Child Health Hospital), and the rest are general hospitals, all participating hospitals have more than 500 beds.

### Study period

The study was from January 1, 2014 to December 31, 2018.

### Inclusion criteria

Patients with CVCs admitted to the participating ICUs or patients insert new CVCs in the participating ICUs between January 1, 2014 and December 31, 2018. Only the subclavian vein, internal jugular vein, and femoral vein were considered great vessels for the purpose of reporting CLABSI in this study.

### Exclusion criteria

Patients stayed in ICU less than 48 h.

### Follow-up periods

Patients were followed till 48 h after discharge/transfer out from the ICU, or the death.

### Active surveillance

Daily surveillance were taken by designated nurses from ICU to record the number of hospitalized patients and the number of patients with CVC every day at 8:00 am. The staffs of infection control department recorded the patients’ demographic details, reason for admission, outcome, information of catheterization and CLABSI, made spot checks and recorded the implementation of precautions.

Hospitals were given the definition of catheter-related bloodstream infection as: a laboratory-confirmed bloodstream infection where CVC was in place on the date of event or the day before. Laboratory-confirmed bloodstream infection criteria were consistent to the United States CDC’s NHSN 2013 surveillance criteria [8].

Since April 1, 2014, the participating hospitals implemented observation, education, performance monitoring, feedback to continuously promote the adherence rates of following intervention:hand hygiene, using full-barrier precautions during the insertion of central venous catheters, cleaning the skin with chlorhexidine, avoiding the femoral site if possible, and removing unnecessary catheters.

Because this is a multi-center cooperative study, it is especially important to ensure the consistency of the surveillance. The surveillance staffs of each hospital were trained before the investigation, about the surveillance procedure, diagnostic criteria and questionnaire filling requirements. Moreover, quality supervisor of each province/municipality would collect and review provincial data every month during the study period to ensure the completeness and credibility of the study. All data were submitted via a uniform data collection tool, with electronic data submission available from the start of the study.

### Statistical analysis

Statistical analysis was performed using SPSS version 25 and website of Open Source Epidemiologic Statistics for Public Health. Medians and interquartile ranges were used for continuous variables. Linear-by-Linear Association was used to compare the trend of annual CLABSI incidences and annual implementation rates of precautions. The two incidences of CLABSI from different regions and ICUs were compared by using Person-time Analysis.

## Results

Total 38,212 patients were admitted to the ICUs with 45,978 CVCs utilized in the study period. The surveillance data were reported over 466,585 catheter days. 702 CLABSI cases occurred, the average CLABSI incidence in a thousand catheter days was 1.50 and the average CVC utilization rate was 42.85%. As shown in Table 1, the participating hospitals and ICUs had changed slightly every year during the study, and total 79 ICUs from 62 hospitals in 11 provinces and municipalities participated in this study.

**Table 1:** Participating intensive care units (ICUs) and number of monitoring patients

Year	No. of participating provinces/municipality	No. of participating hospitals	No. of participating ICUs	No. of monitoring patients
2014	11	41	54	9518
2015	11	57	61	8128
2016	11	47	50	8219
2017	9	33	36	6880
2018	7	23	27	5467

Table 2 showed the characteristics of patients and catheter enrolled in this study. The age of patients ranged between 1 days to 104 years old. Only 37,770 catheters insert in the participated hospital (25,113 catheters from ICUs, and 12,657 from non-ICU departments, such as Emergency Departments, Operating Rooms, etc.). Multi-position catheterization were used in 690 patients (1.81%) which were significantly more frequently seen with CLABSI.

**Table 2:** Characteristics of patients and catheters enrolled in the study

Characteristics	No./$\overline{X}$ ± *S* (*Q*1, *Q*2, *Q*3)
Gender, no.	
Male	24,082
Female	14,130
Age, y	59.94 ± 21.11 (48, 63, 76)
APACHE II score, no.	21.44 ± 9.14 (14, 19, 25)
LOS in ICU before CLABSI, d	10.96 ± 16.61 (3, 6, 12)
Catheter site, no.	
Subclavian	18,141
Jugular	18,230
Femoral	8917
Multi-position catheterization	690
Device days, d	9.82 ± 13.30 (3, 6, 11)

LOS in ICU before CLABSI: patients without CLABSI counts from the day of ICU admission to the day out of ICU

Device days: only catheter insert in ICU and during the ICU admission periods had only one catheter counts (N = 25,360, CLABSI number = 517). Counts from the day of indwell to the day of CLABSI/removal or days of discharged from the ICU

APACHE II score: 12,119 patients’ APACHE II scores were not available due to incomplete data on Acute Physiology Score (APS) items

Multi-position catheterization: there are more than two catheters at the same time

### Rate of CVC utilization and CLABSI incidence

During the study period, the utilization rate of CVC had slowly decreased to about 40% yearly, but the CLABSI incidence in a thousand catheter days had increased to about 1.65 (Table 3). The implementation rates of all precautions showed an upward trend during the study period (P ≤ 0.001) (Table 4).

**Table 3:** Rate of CVC utilization rate, CLABSI incidence and mortality

Year	2014	2015	2016	2017	2018	*P* value for trend test
No. of hospitalization days	271,383	231,437	215,747	205,648	164,707	
No. of device days	120,595	100,367	93,466	86,141	66,016	
No. of patients with CLABSI	159	119	151	164	109	
No. of death with CLABSI	32	31	27	27	10	
Utilization rate of CVCs, % *M* (*Q*1, *Q*2, *Q*3)	44.44 (32.46, 44.28, 58.27)	43.37 (31.42, 45.86, 61.45)	43.32 (32.13, 44.71, 60.57)	41.89 (30.06, 39.47, 53.82)	40.08 (21.29, 35.68, 45.46)	.000
CLABSI rate, per 1000 catheter-days *M* (*Q*1, *Q*2, *Q*3)	1.32 (.00, .87, 1.75)	1.19 (.00, .34, 1.62)	1.62 (.00, .97, 1.67)	1.9 (.39, 1.24, 1.89)	1.65 (.00, 1.00, 1.77)	.000
Mortality, % *M* (*Q*1, *Q*2, *Q*3)	20.13 (.00, 12.12, 33.33)	26.05 (.00, 10.00, 33.33)	17.88 (.00, 16.67, 33.33)	16.46 (.00, .00, 33.33)	9.17 (.00, .00, 22.22)	0.024

Mortality: average mortality rate of CLABSI patients in intensive-care units

*P* values: < 0.05 were considered statistically significant

**Table 4:** Adherence rates of interventions

Interventions	Adherence rates,% *M* (*Q*1, *Q*2, *Q*3)	*P* value for trend test
Hand hygiene compliance	78.68 (66.17, 76.92, 88.72)	.001
Hand hygiene accuracy	87.11 (35.85, 84.29, 93.86)	.000
Using full-barrier precautions	90.76 (86.21, 100, 100)	.000
Cleaning the skin with chlorhexidine	52.73 (0.54, 43.30, 100)	.000
Proportion of femoral site insertion	20.09 (1.36, 9.47, 25.35)	.000
Removing unnecessary catheters	92.58 (96.19, 100, 100)	.000

Adherence rates: average adherence rates for interventions annual

*P* values: < 0.05 were considered statistically significant

Rate of CVC utilization and CLABSI incidence in different kinds of ICU were shown in Table 5. The department with the lowest CVC utilization rate was pediatric ICU, the department with the highest CVC utilization rate was surgical ICU, and there was no infection case monitored in pediatric ICU. The lowest and highest CLABSI incidence in different kind of ICU was pediatric ICU (0/1000 catheter days) and cardiac ICU (2.48/1000 catheter days), respectively.

**Table 5:** Rate of CVC utilization and CLABSI incidence in different ICUs

ICU type	No. of hospitalization days	No. of device days	No. of patients with CLABSI	Utilization rate of CVCs,% *M* (*Q*1, *Q*2, *Q*3)	CLABSI rate, per 1000 catheter-days *M* (*Q*1, *Q*2, *Q*3)	*P* value
General ICU	916,504	393,391	649	42.92 (27.78, 44.60, 55.57)	1.65 (.32, 1.07, 2.74)	
Pediatric ICU	3964	232	0	5.85 (2.37, 3.11, 7.93)	.00 (.00, .00, .00)	.5361
Emergency ICU	23,687	9810	3	41.42 (18.43, 41.85, 49.19)	.31 (.00, .00, .00)	.001
Medical ICU	55,520	26,242	9	47.27 (11.39, 44.65, 58.46)	.34 (.00, .00, .39)	.000
Neurological ICU	20,490	7274	6	35.50 (27.67, 31.16, 34.04)	.82 (.00, .00, 1.04)	.0847
Respiratory ICU	37,606	12,794	12	34.02 (13.32, 31.21, 47.80)	.94 (.00, .00, .69)	.0495
Surgical ICU	25,321	16,439	22	64.92 (32.33, 58.71, 70.77)	1.34 (.00, .61, 1.94)	.3336
Coronary Care Unit	5830	403	1	6.91 (3.11, 5.11, 6.98)	2.48 (.00, 1.25, 3.86)	.6813

*P* value: CLABSI incidence of Special ICUs were separately compared with General ICU. *P* values < 0.00625(0.05/8 = 0.00625) were considered statistically significant

Rate of CVC utilization and CLABSI incidence in different areas of China were shown in Table 6. The area with the lowest CLABSI incidence and CVC utilization rate was Northeast China (0.77/1000 catheter days) and Central China (38.05%), respectively, and the highest CLABSI incidence and CVC utilization rate during the study period was Eastern China (1.62/1000 catheter days) and Western China (51.57%), respectively.

**Table 6:** Rate of CVC utilization and CLABSI incidence in different areas of China

Region of China	No. of hospitalization days	No. of device days	No. of patients with CLABSI	Utilization rate of CVCs,% M (Q1, Q2, Q3)	CLABSI rate, per 1000 catheter-days M (Q1, Q2, Q3)	*P* value
Central China	230,783	87,818	120	38.05 (29.94, 38.29, 54.83)	1.37 (.00, .52, 1.60)	
Eastern China	667,988	285,205	462	42.7 (28.47, 45.25, 60.24)	1.62 (.00, 1.00, 2.57)	.0964
Northeast China	64,038	28,522	22	44.54 (36.83, 37.15, 45.09)	.77 (.00, .62, .91)	.0095
Western China	126,113	65,040	98	51.57 (37.13, 63.82, 84.78)	1.51 (.00, .91, 1.73)	.4728

Region of China: according to the situation of China’s economic and social development, the country is divided into four major regions. Northeast China: Liaoning; Central China: Shanxi, Henan, Hunan; Eastern China: Beijing, Jiangsu, Zhejiang, Shandong, Guangdong; Western China: Guizhou, Sichuan

*P* value: CLABSI incidence of different regions of China were separately compared with Central China. *P* values < 0.0125(0.05/4 = 0.0125) were considered statistically significant

### Microbiology and susceptibility patterns

Among 702 CLABSI cases reported in the ICUs, 735 pathogens were isolated. Table 7 showed the distribution of identified pathogens and their resistance patterns. *Staphylococcus* spp. was the most common organism isolated (including 33 strains of *S. aureus*), accounting for 27.07% of all CLABSI cases, followed by *Enterobacteriaceae* (22.31%). 66.67% of *S. aureus* were methicillin-resistant *S. aureus* (**MRSA**), and 33.54% of *Enterobacteriaceae* were resistant to carbapenem.

**Table 7:** Distribution of pathogens associated with CLBASI in ICUs and their pattern of antibiotic resistance

Pathogen identified and pattern of antibiotic resistance	No. of CLABSI cases (%) (n = 735)	No. of resistance (%)
*Enterococcus* spp.	71 (9.66)	
Vancomycin-resistant Enterococcus		13 (18.31)
*Acinetobacter* spp.	144 (19.59)	
Carbapenem resistant		68 (47.22)
*Pseudomonas* spp.	40 (5.44)	
Multidrug resistant		23 (57.50)
*Staphylococcus* spp.	199 (27.07)	
MRSA		22 (66.67)
*Enterobacteriaceae*	164 (22.31)	
Carbapenem resistant		55 (33.54)
*Streptococcus* spp.	8 (1.09)	
*Stenotrophomonas maltophilia*	5 (0.68)	
Fungus	89 (12.11)	
Other Gram-positive cocci	3 (0.41)	
Other Gram-positive bacilli	3 (0.41)	
Other Gram-negative bacilli	9 (1.22)	

## Discussion

Since 2007, health care-associated infection surveillance of ICU has been carried out gradually throughout China. Especially after the publication of the “Standard for nosocomial infection surveillance [9]” issued by the Ministry of Public Health in 2009, targeted surveillance has become the main direction of health care-associated infection surveillance in China. The surveillance of CLABSI is limited in ICU department, and the surveillance catheters were mainly CVCs, peripherally inserted central venous catheters (PICC) and umbilical vascular catheters.

The different definitions of surveillance, diagnostic criteria, patient conditions, and catheterization procedures in different countries and regions may lead to the differences in the incidence of CLABSI. In the United States, based on surveillance data of 2014 by the United States Medical Safety Network (NHSN) released in 2016, the standardized infection rate (SIR) for CLABSI was 0.5/1000 catheter days [10]. A surveillance of CLABSI incidence in ICUs of four European countries showed that CLABSI incidence was 4.2/1000 catheter days in UK, 1.5/1000 catheter days in Germany, 2.0/1000 catheter days in Italy, and 1.23/1000 catheter days in France [11]. 384 CLABSI events were reported over 303,968 CVC days, corresponding to a rate of 1.26/1000 catheter days (95% confidence interval, 1.14–1.40) in Australian [12]. Studies in 18 developing countries showed that CLABSI incidence was around 6.9–15.2 per 1000 catheter days [13]. In this study, CLABSI incidence was comparable to some developed countries, but was significantly lower than developing countries including China [14]. Since most of our data were collected from tertiary care and general hospitals with more than 500 beds (only three secondary care hospitals) in 11 provinces and municipality, our results reflected the current incidence of CLABSI and adherence rates of interventions in large general hospitals in China.

The average utilization rate of CVC in this study was lower than that reported in 140 hospitals of Jiangsu province in China (52.27%) [15] and that reported in Germany (82.2%) [16]. The difference may be related to the fact that we only included central line inserted in internal jugular vein, subclavian vein, and femoral vein, but excluded central line inserted in other venous and arterial such as implantable venous access port or PICC. It can also be seen from our results that the utilization rate of CVC of different ICUs were quite different, and our study showed a low percentage of surgical ICU which had higher utilization rate of CVC.

Although we have developed detailed protocols to reduce the incidence of CLABSI, CLABSI incidence had no significant decrease during the study period, but the utilization rate of CVC had slowly decreased to about 40%. From the 3rd year of the study, the research group adjusted the types of participating ICU with focus on general ICU, medical ICU and respiratory ICU, which had relatively high utilization rate and high CLABSI incidence. This may explain the fluctuations of CLABSI incidence annually.

Pediatric ICU had the lowest utilization rate because pediatrics choose PICC as the main way of infusion, and there was no infection case for short surveillance time, so the infection rate was the lowest. In contrast, most patients in surgical ICU need CVCs due to surgery, leading to the highest utilization rate. Among different ICUs, general ICU had the maximum sample size and the most stable surveillance source, so the data were more reliable. CLABSI incidence of general ICU was the closest to the average incidence because the patients came from various specialist departments and had more complicated condition. Other ICUs such as emergency ICU, medical ICU, respiratory ICU and neurological ICU had relatively lower incidences of CLABSI.

In addition, CVC utilization rate and CLABSI incidence showed differences across different regions of China. Infections tend to occur in the eastern part of the country, with relatively fewer infections in the northeast part. Due to current significant imbalance in medical and health resources, some patients, especially the critically ill, will choose to go to a large hospital in the eastern region for medical treatment. This may increase the number of CLABSI cases in the eastern region to some extent. The CVC utilization rate in Western part of China was higher than in other regions, and the rate in some hospitals and ICUs was even as high as 90%, indicating that some hospitals have placed CVCs for patients who do not meet the indications. Nevertheless, the differences between regions cannot be explained only by the aforementioned parameters, and need more detailed studies.

We also investigated the pathogenic microorganisms and their drug resistance associated with CLABSI cases. Studies have shown that the main pathogens of CLABSI are coagulase-negative *Staphylococcus*, *S aureus*, *enterococcus*, and candida due to the adhesion to the epidermis and wounds [6, 12, 17–20]. However, recent studies have found that the incidence of CLABSI caused by Gram-negative bacilli is increasing [7, 21–25]. The results of this study showed that Gram-negative bacteria account for a greater proportion (49.24%) than Gram-positive bacteria (38.17%) in the CLABSI cases, and there is an upward trend. Participating hospitals have adopted CLABSI preventive measures, such as cluster care strategies, which have reduced the adhesion of Gram-positive bacteria to the epidermis and wounds. This may explain the decline in the proportion of Gram-positive bacteria in CLABSI pathogens.

As a morbidity survey, our study has several limitations. First, the areas covered by this study is not complete. There are 34 provinces, municipalities and autonomous regions in China, and our study only covered 11 of them. In particular, the coverage of the western and northeastern regions is not enough, such as Xinjiang and Tibet, which are precisely the weakest areas of health care-associated infection surveillance in China. Second, the hospitals participating in the surveillance in various provinces/municipality were not randomly selected, but were selected by voluntary registration. There is also no requirement on the number of participating hospitals and ICUs in each province, resulting in a small number of participating hospitals and ICUs in some provinces. Third, because there is no reporting software system, the accuracy of data cannot be actively controlled and screened, which increases the workload of manual reporting and review. Therefore, during the surveillance process, some hospitals withdrew from the surveillance group due to insufficient quality of data reporting or heavy reporting workload. Despite these limitations, our data represent the trend of CLABSI epidemiology in 2014–2018 for most tertiary general public hospitals in China.

## Conclusion

The average incidence of CLABSI in this study was 1.5/1000 catheter days, lower than previous reports in China, but similar to the rate reported in developed countries. As a continuous surveillance project, targeted surveillance of ICU CLABSI cases can not only detect the changing trend of incidence and pathogens, but also find the regional differences in CLABSI incidence. It is necessary to implement targeted surveillance of CLABSI cases by using standardized CLABSI surveillance definitions and methodologies.

## Data Availability

The data from our surveillance are not available on the public domain, but anyone interested in using the data for scientific purpose is free to request permission from the corresponding author: Anhua Wu (xywuanhua@csu.edu.cn).

## Abbreviations

ICU: Intensive care unit
CLABSI: Central line-associated bloodstream infection
CVC: Central venous catheter
CDC: Centers for Disease Control and Prevention (America)
NHSN: National Healthcare Safety Network (America)
PICC: Peripherally inserted central venous catheter
SIR: Standardized infection rate
